# Development of Vision Based Multiview Gait Recognition System with MMUGait Database

**DOI:** 10.1155/2014/376569

**Published:** 2014-03-27

**Authors:** Hu Ng, Wooi-Haw Tan, Junaidi Abdullah, Hau-Lee Tong

**Affiliations:** Faculty of Computing and Informatics, Multimedia University, 63100 Cyberjaya, Malaysia

## Abstract

This paper describes the acquisition setup and development of a new gait database, MMUGait. This database consists of 82 subjects walking under normal condition and 19 subjects walking with 11 covariate factors, which were captured under two views. This paper also proposes a multiview model-based gait recognition system with joint detection approach that performs well under different walking trajectories and covariate factors, which include self-occluded or external occluded silhouettes. In the proposed system, the process begins by enhancing the human silhouette to remove the artifacts. Next, the width and height of the body are obtained. Subsequently, the joint angular trajectories are determined once the body joints are automatically detected. Lastly, crotch height and step-size of the walking subject are determined. The extracted features are smoothened by Gaussian filter to eliminate the effect of outliers. The extracted features are normalized with linear scaling, which is followed by feature selection prior to the classification process. The classification experiments carried out on MMUGait database were benchmarked against the SOTON Small DB from University of Southampton. Results showed correct classification rate above 90% for all the databases. The proposed approach is found to outperform other approaches on SOTON Small DB in most cases.

## 1. Introduction

Biometrics is a way to identify individuals through their physical and behavioral characteristics such as fingerprint, gait, face, iris, and spoken speech. These characteristics are known as biometric identifiers that are distinctive and will be attached to a person permanently. Gait is a biometric modality that gained public recognition and is well accepted as security assessment. This is mainly because the user does not require any contact or intervention with the capturing device. Furthermore, it is still capable of identifying people at a distance even if other biometrics identifiers are intentionally obscured (hand gloves to cover finger print and mask to cover face).

The development of gait database has started since 1998 with the University of California San Diego (UCSD) gait database [1] which consists of six subjects with a total of 42 outdoor walking sequences. In 2002, the University of Southampton released the SOTON gait database [2] that contains the Large DB with 115 subjects and the Small DB with 11 subjects involving 15 covariate factors. The HumanID Gait Challenge [3] was released in 2005. It comprised 122 subjects with five major covariate factors (surface, shoes, carrying, camera angles, and time). In 2005, CASIA database B [4] was published with three covariate factors (bag, normal, and coat) with 11 different view angles. In 2012, the Treadmill Dataset B from OU-ISIR Gait Database [5] provided 64 subjects with 32 combinations of clothing variations while user was walking on treadmill. However, Lee and Hidler [6] demonstrated that there are differences in optical flow between subjects walking on treadmill and solid ground. As such, this database may not be suitable for gait recognition evaluation as it does not reflect real world scenarios.

While many gait databases are available, we argue that all of them have neglected one important challenge: none of the male subjects are wearing long fabrics covering the legs. Most of the databases contain male subjects that are only wearing trousers. The closest breakthrough is the SOTON database, where there are two female subjects (out of 115 subjects) wearing long blouse and Indian traditional garment (“salwar kameez”). On the other hand, OU-ISIR Treadmill Database B contains short skirt as a covariate factor for the participants. However, the short skirt is only partially covering the legs with the knees still clearly visible. In reality, males do wear long fabrics such as “sarong” and “kain samping” for ethnic Malays in South East Asia, long “dhoti” in Southern Asia, and “kilt” in Scotland. Due to this reason, it motivates us to build database with subjects wearing “sarong” and “kain samping”.

Our database consists of 82 subjects on normal condition for reliable performance evaluation on the large population and 19 subjects with 11 covariate factors as emphasis for the evaluation on the exploratory factor analysis of gait recognition. It contains side-view and oblique-view videos, the extracted silhouette frames, subject still face images, and ancillary data like subject information, cameras setup, and floor measurements.

We also propose a multiview gait recognition system, tested on this database, and benchmarked with the SOTON Small DB. This system comprises database construction, viewpoint normalization, gait feature extraction, feature processing, and classification. In the next Section, we present existing research works on gait recognition.

## 2. Related Work

Various research works have been conducted in gait recognition system. This section reviews the related literature on gait features extraction approaches and multi-view normalization approaches.

### 2.1. Gait Features Extraction Review

Generally, the approaches on gait features extraction are divided into two major groupings, which are model-based approaches and model-free approaches. Model-based approaches [7, 8] normally imitate the human body structure as two dimensional boxes. Then, it integrates with the knowledge of the body shape and match as model components. The capability to acquire the dynamic gait features from model parameters is the main advantage of these approaches. It is competent to liberate the background noise and is also able to avoid the bad effects from the changes of the camera view-points or subject's apparel. Conversely, it generates complex model during the extraction process, which required high computational power, massive storage space, and huge experimental cost.

On the other hand, model-free approaches [9–12] simplify the entire human body to a concise representation using silhouette or skeleton. The significant advantages of these approaches are that they are related straightforward and only require minimum memory usage with low computational cost. Nevertheless, their performance is heavily defected by the background noise and the variations of the subject's apparel.

In this paper, we employed model-based approach for the extraction of gait features. The approach is based on human body proportion [13], which is similar to the work of Goffredo et al. [10] and Yoo and Nixon [12]. Compared to their work, our approach is related more straightforward as it does not involve the measurement of gait cycle for kinematic parameter calculation. Moreover, manually labelling of the model template is not required, as performed by Goffredo et al.

### 2.2. Multiview Normalization Review

There are three major types of approaches that solved the issues in the multiview normalization, namely, view invariant gait feature, view synthesis, and view transformation.

In the first approach [14–17], they are able to extract gait features that are invariant to changes in walking trajectory and camera view-point. However, these approaches can only be applied to a few limited viewing angles and their feature extraction processes can disrupted due to occlusion.

In the second approach [18, 19], they reconstruct gait motion by 3D information from calibrated multiple view-point cameras. They manage to generate precise synthetic images, but it requires heavy computational resource and complicated technical setup due to camera calibration.

In the third approach [20, 21], they reconstruct the gait features into the same view by mapping the relation between the gait features with the subject. However, these approaches propagate numerous noises during the reconstruction process, which degrades the recognition performance.

We employ perspective correction for view invariant gait feature extraction, which is comparable to Jean et al. [15]. However, we do not extract the spatiotemporal trajectories of body parts for gait modeling. We managed to mitigate the problems with missing heads or feet in the silhouettes as faced by Jean et al. In addition, our approach does not require the detection of half gait cycle as proposed by in Jean et al. We also do not attempt to detect each of the lower limbs. Thus, it can handle occluded silhouette either from self-occlusion or those occluded by apparels, which are normally disastrous for other view invariant gait feature approaches.

Our approach is also free from view synthesizing and camera calibration processes and is related more straightforward and faster than the view synthesis and view transformation approaches.

## 3. Methodology 

According to Murray [22], it is not practical to measure pelvic and thorax rotations. Furthermore, they were found to be inconsistent after repeated tests. Thus, we only consider gait features from the lower limbs. Nonetheless, our system does not attempt to detect each of the lower limbs. Therefore, it can handle occluded silhouette which may be either due to self-occlusion or external occlusion, such as occlusion by subject's apparel (long blouses or baggy trousers) or load carrying. These conditions are normally disastrous for model-free approaches. It is also straightforward, faster, and simpler than other model-free approaches.

**Figure 1:**
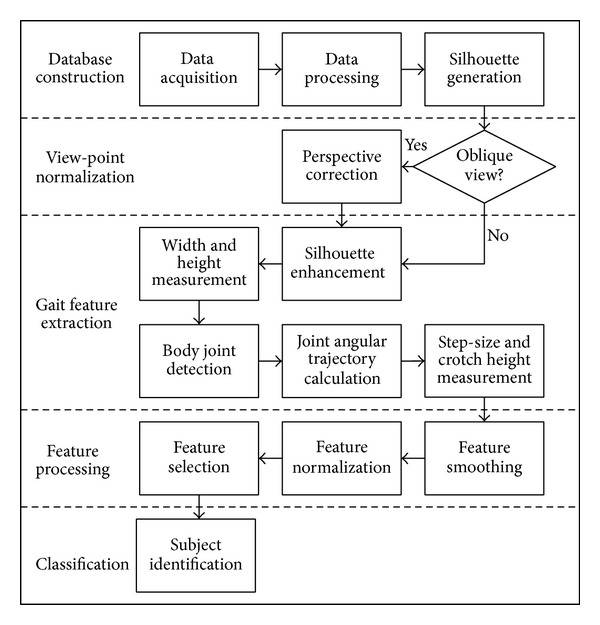
Proposed processes flowchart.

In view of the fact that the static body parameters and the dynamics of human walking stance are the main structures in gait configuration [22], we present a model-based approach to extract subject's height, width, step-size, and crotch height as the static features and compute joint angular trajectories as the dynamic features.  Figure 1 illustrates the flow of the processes.

### 3.1. Acquisition of the MMUGait Database

**Figure 2:**
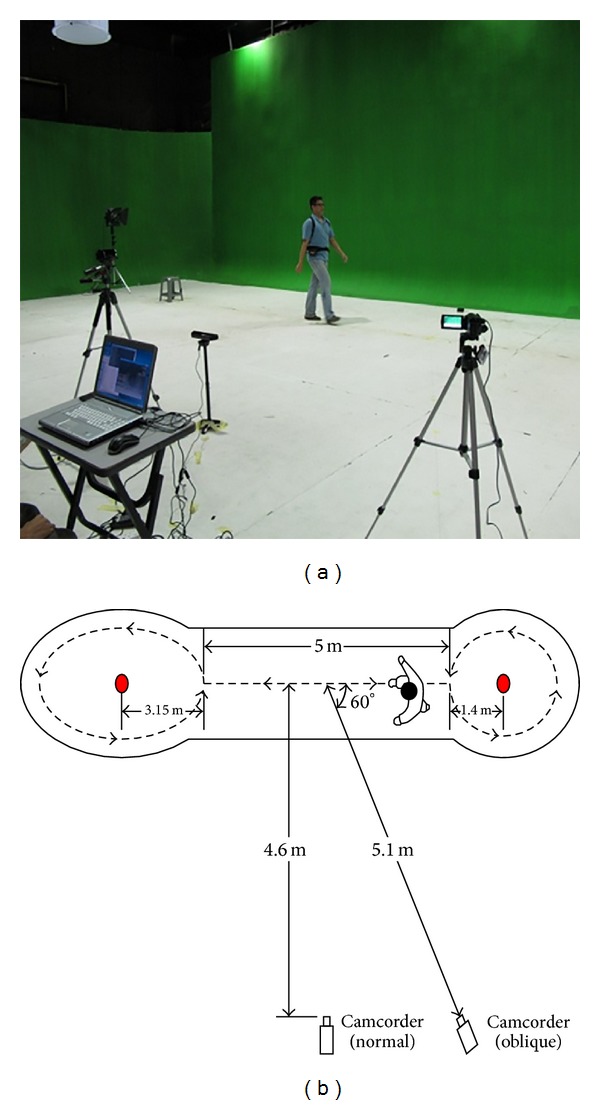
(a) The acquisition environment. (b) The recording layout.

The MMUGait database is part of the MMU GASPFA database [23]. It was captured and recorded in the Set and Background Studio located in the Faculty of Creative Multimedia, Multimedia University. The acquisition was done over a period of four days in December 2011 with involving 82 participants. Ethical approval was obtained from the participants by signing a standard university approval consent form prior to volunteering. The recording of MMUGait database was done in an indoor environment with green backdrop and white solid surface.  Figure 2(a) shows the acquisition environment.

Two SONY HDR-XR160E full HD video camera recorders (camcorders) were used during the filming. The recorded videos are in MPEG Transport Stream (MTS) format with resolution of 1920 (Height) × 1080 (Width) pixels. The video stream was captured using progressive scan with a frame rate of 50 frames per seconds (fps). The camcorders captured the walking sequence from two different view angles, which were side-view (frontal parallel) and 60° oblique-view.

The subjects walked back and forth on a track continuously and captured in both directions. Both ends of the walking track were the turning point that subjects must turn around and repeat the walking sequence in another direction.  Figure 2(b) shows the recording layout.

There were 82 participants that took part in the recording of the MMUGait database. They were from different nationalities, genders, and age groups. There were 67 subjects from Malaysia, while the remaining 15 subjects came from six other countries (the Middle East, Central Asia, and Africa). For the gender class, 15 subjects were female, while the remaining 67 were male. 71 subjects were aged between 15 and 25 years, while the rest fell between the ages of 26–40 years.

There are two categories of database collected in this phase. The first set (MMUGait Large DB) contains 82 subjects that walked for at least 20 sequences in both directions with personal clothing (own shoes and own cloth). On the other hand, the second set (MMUGait Covariate DB) contains 19 subjects that walked in both directions wearing different types of clothes, shoes, and carrying various types of bags, with varying walking speed. This set includes 11 covariant factors, which are “sarong”, “kain samping”, personal clothing, hand bag (held in hand), barrel bag (slung over shoulder), barrel bag (carried by hand), rucksack, walking slowly, walking quickly, walking in flip flops, and bare feet. In total, there were approximately 110 sequences per subject.

To the best of our knowledge, this is the first paper that introduces biometric gait recognition with Malays' traditional costumes, which are common attire for ethnic Malays in South East Asia, especially during Friday prayers and religious festivals. In this case, we recorded subjects with “kain samping” and “sarong” as covariate factors as changes in apparel. We believe that it can act as a benchmark database for performance evaluation by other gait recognition systems.

### 3.2. Data Processing

The original video format filmed was in MPEG transport stream format (MTS) with 50 frames per second (FPS). For further processing, all the recorded videos were converted to Audio Video Interleave (AVI) format with resolution of 1920 (High) ∗ 1080 (Width) pixels. After that, the videos were extracted into individual frames in Joint Photographic Experts Group (JPEG) format.

### 3.3. Silhouette Generation

To extract the human silhouette from each extracted frame, background subtraction technique was employed. We have improved the conventional background subtraction technique by summing up the gray level results from (a) background image subtracted by foreground image and (b) foreground image subtracted by background image. This approach is able to amplify the difference between the foreground and the background. As a result, the foreground object is more distinctive.

Next, the pixels' intensity was adjusted to increase the contrast of the foreground object. The process was carried out by obtaining optimal thresholding via Otsu's method [24]. Once the threshold was found, it was used to rescale the gray level values to new values such that values between the lowest input value and the threshold were scaled to values between 0 and 1.

By applying the Otsu's method [24] again, the gray scale image was then converted to binary image using the new threshold. In this case, if a pixel value was below the threshold, it was set to zero; otherwise, it was set to one in the binary image.

After that, the morphological operations with a 7 × 7 diamond shape structuring element are applied to enhance the generated foreground object. Morphological opening was applied to separate the shadow into isolated regions, while morphological closing is used to close the small gaps in the foreground object.

**Figure 3:**
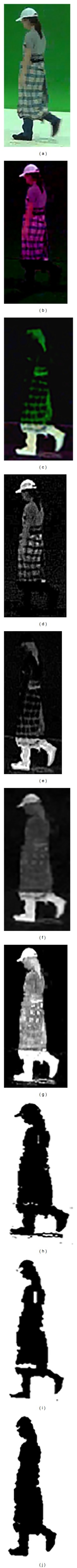
(a) Foreground image. (b) Color image of foreground subtracts background. (c) Color image of background subtracts foreground. (d) Gray level image of (b). (e) Gray level of (c). (f) Addition of (d) and (e). (g) Foreground object after intensity adjustment. (h) Binary image of foreground object. (i) Foreground object after morphological opening. (j) Extracted silhouette.

In some cases, noise still exists as pseudoobjects in the image. Connected component labeling is applied to label all regions within the image. Subsequently, those regions with area smaller than 1500 pixels are removed.  Figure 3 shows the resulting images at different stages of silhouette generation.

The pseudocodes of the processes involved are described below.

*Definition of Notations*

 
$\vec{p}=(x,y)$: running pixel;
 
*D* = {(*x*, *y*) | 0 ≤ *x* < *N*_*x*_, 0 ≤ *y* < *N*_*y*_}: domain of image;
 
*B*(*x*, *y*): background image;
 
*F*(*x*, *y*): foreground image;
 Δ_1_(*x*, *y*): foreground object with pixels of higher intensity than background image;
 Δ_2_(*x*, *y*): foreground object with pixels of lower intensity than background image;
 Δ(*x*, *y*): foreground object in gray scale;
 
*T*_1_, *T*_2_: thresholds generated from Otsu's method;
 Δ′(*x*, *y*): foreground object after contrast enhancement;
 
*C*(*x*, *y*): binary image of foreground object;
 
*D*_1_(*x*, *y*): binary image after morphological opening with structuring element *S*;
 
*D*_2_(*x*, *y*): binary image after morphological closing with structuring element *S*;
 
*E*(*x*, *y*): labeled image after connected component labeling;
 
*I*(*x*, *y*): labeled image after blobs removal;
 trun(): truncate negative pixel values to zero;
 rgb2gray(): convert RGB values to gray level values;
 Ostu(): Ostu's thresholding technique;
 scale(): rescale the gray level values from 0 and *T*_1_ to 0 and 1, respectively;
 concom(): connected component labeling;
 blobremove(): remove blobs with size smaller than *r*;

*Pseudocode*

$\forall \vec{p}\in D$

 Δ_1_($\vec{p}$) = trun $\left(F(\vec{p})-B(\vec{p})\right)$
 Δ_2_($\vec{p}$) = trun $\left(B(\vec{p})-F(\vec{p})\right)$
 Δ($\vec{p}$) = rgb2gray (Δ_1_($\vec{p}$)) + rgb2gray (Δ_2_($\vec{p}$))

*T*_1_ = Ostu(Δ)

$\forall \vec{p}\in D$

 
$\Delta \prime (\vec{p})=\text{scale}(\Delta (\vec{p}),0,T_{1})$

*T*_2_ = Ostu(Δ′)

$\forall \vec{p}\in D$

 
  (1) $$\begin{array}{c} C\left(\vec{p}\right)=\{\begin{array}{cc} 1, & \Delta^{\prime }\left(\vec{p}\right)>T_{2}\\ 0, & \Delta^{\prime }\left(\vec{p}\right)\leq T_{2} \end{array} \end{array}$$

*D*_1_ = (*C* ⊖ *S*) ⊕ *S*

*D*_2_ = (*D*_1_ ⊕ *S*) ⊖ *S*

*E* = concom(*D*_2_)

*I* = blobremove(*E*, *r*). 

### 3.4. View-Point Normalization

**Figure 4:**
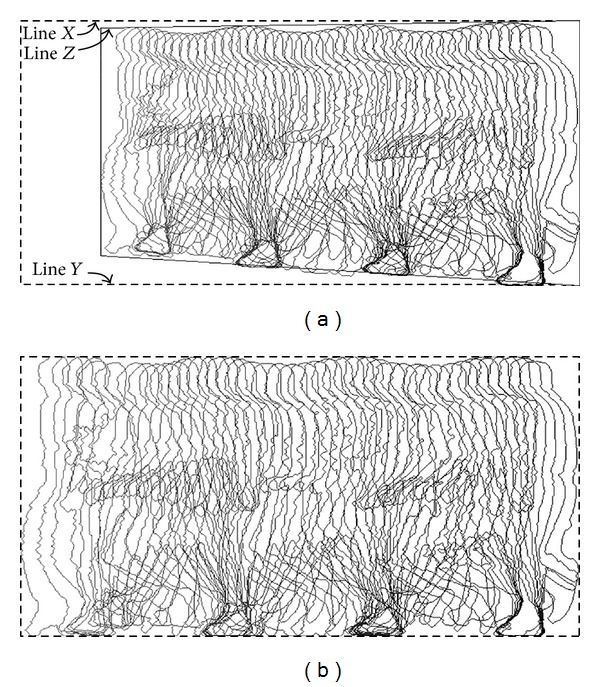
(a) Superimposed silhouettes from one walking sequence. (b) Superimposed silhouettes after perspective correction.

To normalize the oblique walking sequence into the side-view plane, the perspective correction technique is employed. First, all silhouettes in a walking sequence are superimposed into a single image, as shown in Figure 4(a).

Next, lines* X* and* Y* are drawn horizontally based on the highest and lowest point among the silhouettes. As the normal gait cycle is periodic, a sinusoidal line is formed when the highest points of all silhouettes in a walking sequence are connected. Line* Z* is then drawn by connecting the first peak and the last peak of the sinusoidal line.

The perspective correction technique consists of two stages: vertical and horizontal adjustments. For vertical adjustment, each silhouette is vertically stretched from line* Z* towards line* X*. In addition, each silhouette is also vertically stretched from the bottom towards line* Y*.  Figure 4(b) shows superimposed silhouettes after perspective correction.

### 3.5. Feature Extraction

Several processes were involved in the extraction of the gait features from the human silhouette images. The details of these processes are discussed in the following subsections.

#### 3.5.1. Enhancement of Silhouettes

Occasionally, the foreground object segmentation does not work well due to noise. As a result, the boundaries of the segmented human silhouette are coarse with jagged edges. Sometime, spurious regions may also present near the border of the human silhouette or even in the background. If horizontal lines are drawn across the human silhouette for automatic body joint identification, the jagged edges and spurious regions will hinder the process as they may be erroneously considered as parts of the human silhouette.

**Figure 5:**
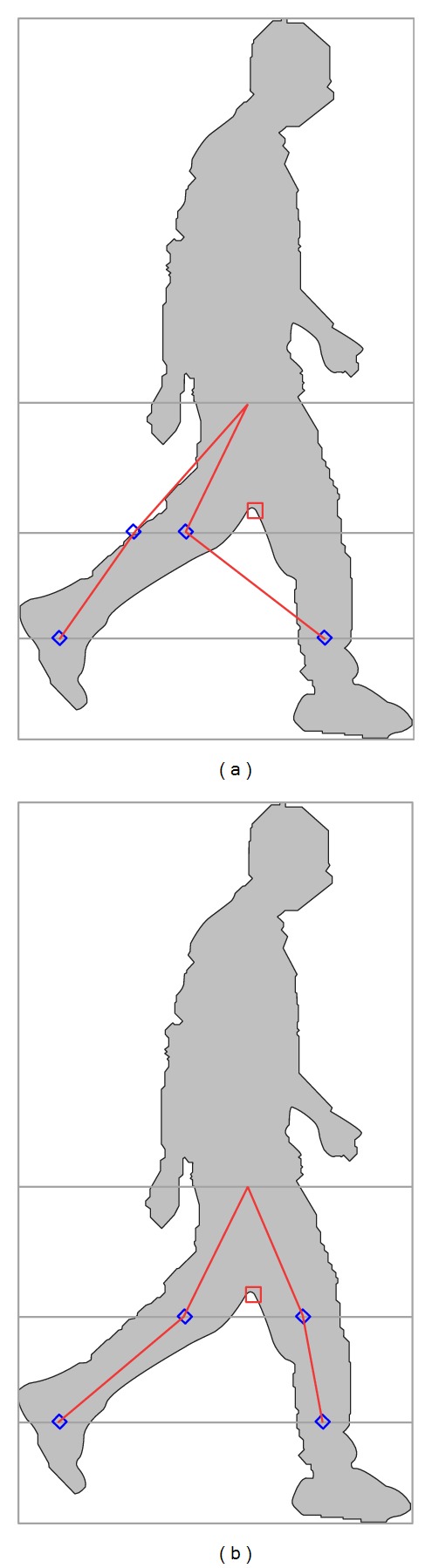
(a) Results without jagged edge exclusion. (b) Results with jagged edge exclusion.

To mitigate these problems, the width of the regions crossed by the horizontal lines should be determined. To determine the width of these regions, the distance between the rising edge and falling edge of a region is determined. If the width of a region is less than a threshold value, that region is considered as invalid and will be excluded during the body joint identification process. In the proposed technique, the threshold value has been empirically determined as five percent of the human silhouette height.  Figure 5 shows the improvement on joint detection with jagged edge exclusion. In Figure 5(a), there is a jagged edge at the left side of the back knee. When the proposed body joint identification technique draws a horizontal line across the human silhouette at knee height, the horizontal line crosses the jagged edge as well as both knees. The horizontal line is then scanned from left to right and the middle points of the first two regions that it crosses are identified as the center of the knees. In this example, the jagged edge has been erroneously identified as the center of a knee while the actual center of the knee at the front leg has been completely ignored even though the jagged edge is only a few pixels wide. By excluding the regions narrower than the threshold value, the jagged edge has been excluded from the body joint identification process and the centers of both knees have been correctly identified as shown in Figure 5(b).

#### 3.5.2. Bounding Box Measurement

**Figure 6:**
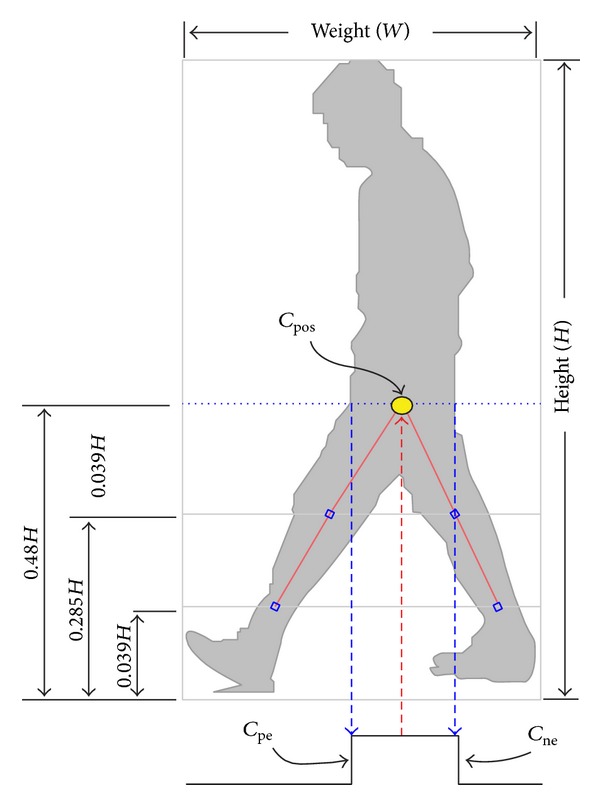
The height and width of a human silhouette with the image profile of horizontal line drawn through the hip.

After that, the height (*H*) and width (*W*) of the body are obtained from the bounding box of the improved silhouette.  Figure 6 shows the two extracted gait features.

#### 3.5.3. Hip Joint Estimation

By referring to a priori knowledge of the body proportions [13], the vertical position of hip, knee, and ankle are estimated as 0.48*H*, 0.285*H*, and 0.039*H* with referring to the body height *H*. Once these are known, the horizontal position of these body joints can be determined.  Figure 6 shows a horizontal dashed line that goes through the hip with the resulting image profile shown below the silhouette. The horizontal center position of the hip can be found by determining the midpoint between the positive and negative edge by applying the equation below:

(2) $$\begin{array}{c} c_{\text{pos}}=c_{\text{pe}}+\frac{c_{\text{ne}}-c_{\text{pe}}}{2}, \end{array}$$

where *c*_pe_ is the horizontal position of the positive edge, *c*_ne_ is the horizontal position of the negative edge, and *c*_pos_ is the horizontal center position of the hip.

#### 3.5.4. Knee and Ankle Joints Estimation for Nonoccluded Silhouettes

**Figure 7:**
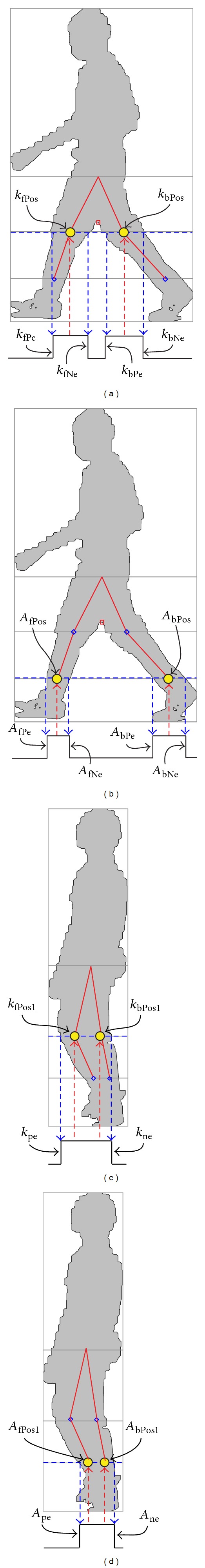
(a) Knee positions on normal silhouette. (b) Ankle positions on normal silhouette. (c) Knee positions on self-occluded silhouette. (d) Ankle positions on self-occluded silhouette.

To locate the center horizontal positions of the knees, a horizontal line is outlined at knee height all the way through the silhouette. The silhouette without self or external occlusion, four edges can be found on the image profile along this horizontal line, as indicated by two dots beside each knee in Figure 7(a). The horizontal center knee positions are discovered by computing the midpoint flanked by two adjacent edges on each knee by the equations below:

(3) $$\begin{array}{c} k_{\text{fPos}}=k_{\text{fPe}}+\frac{k_{\text{fNe}}-k_{\text{fPe}}}{2},\\ k_{\text{bPos}}=k_{\text{bPe}}+\frac{k_{\text{bNe}}-k_{\text{bPe}}}{2}, \end{array}$$

where *k*_fPos_ and *k*_bPos_ are the horizontal center positions of the front and back knee for normal silhouette, *k*_fPe_ and *k*_bPe_ are the horizontal positions of the positive edge on the front and back knee, and *k*_fNe_ and *k*_bNe_ are the horizontal positions of the negative edge on the front and back knee.

To locate the horizontal center position of the ankles, the same method is applied. If a horizontal line is outlined at ankle height, four edges are found on the image profile along the horizontal line on a normal silhouette, as illustrated in Figure 7(b). As a result, the horizontal center ankle positions will be detected by employing the subsequent equations

(4) $$\begin{array}{c} A_{\text{fPos}}=A_{\text{fPe}}+\frac{A_{\text{fNe}}-A_{\text{fPe}}}{2},\\ A_{\text{bPos}}=A_{\text{bPe}}+\frac{A_{\text{bNe}}-A_{\text{bPe}}}{2}, \end{array}$$

where *A*_fPos_ and *A*_bPos_ are the horizontal center positions of the front and back ankle for normal silhouette, *A*_fPe_ and *A*_bPe_ are the positive edge on the front and back ankle, and *A*_fNe_ and *A*_bNe_ are the negative edge on the front and back ankle.

#### 3.5.5. Knee and Ankle Joints Estimation for Occluded Silhouettes

For occluded silhouettes, the horizontal center knee positions are located by determining the midpoint between each edge with referring to the horizontal center position of the hip (as only two edges can be found on the image profile), as shown in Figure 7(c). Consider

(5) $$\begin{array}{c} k_{\text{fPos}1}=k_{\text{pe}}+\frac{c_{\text{pos}}-k_{\text{pe}}}{2},\\ k_{\text{bPos}1}=c_{\text{pos}}+\frac{k_{\text{ne}}-c_{\text{pos}}}{2}, \end{array}$$

where *k*_fPos1_ and *k*_bPos1_ are the horizontal center positions of the front and back knee for occluded silhouette, *c*_pos_ is the horizontal center position of the hip, *k*_pe_ is the horizontal position of the positive edge, and *k*_ne_ is the horizontal position of the negative edge on the corresponding image profile.

Since there are only two edges on the image profile, as highlighted in Figure 7(d), the horizontal center ankle positions are found by determining the midpoint flanked by both edges with employing the equations as

(6) $$\begin{array}{c} A_{\text{fPos}1}=A_{\text{pe}}+0.25\left(A_{\text{ne}}-A_{\text{pe}}\right),\\ A_{\text{bPos}1}=A_{\text{pe}}+0.75\left(A_{\text{ne}}-A_{\text{pe}}\right), \end{array}$$

where *A*_fPos1_ and *A*_bPos1_ are the horizontal center positions of the front and back ankle for occluded silhouette, *A*_pe_ and *A*_ne_ are the horizontal positions of the positive and negative edge on the image profile, and 0.25 and 0.75 are chosen to compute the first quarter and third quarter points between these edges. *C*_pos_ is not used in the formulation as it does not reflect the middle point between *A*_pe_ and *A*_ne_.

#### 3.5.6. Joint Angular Trajectory, Step-Size, and Crotch Height Calculation

**Figure 8:**
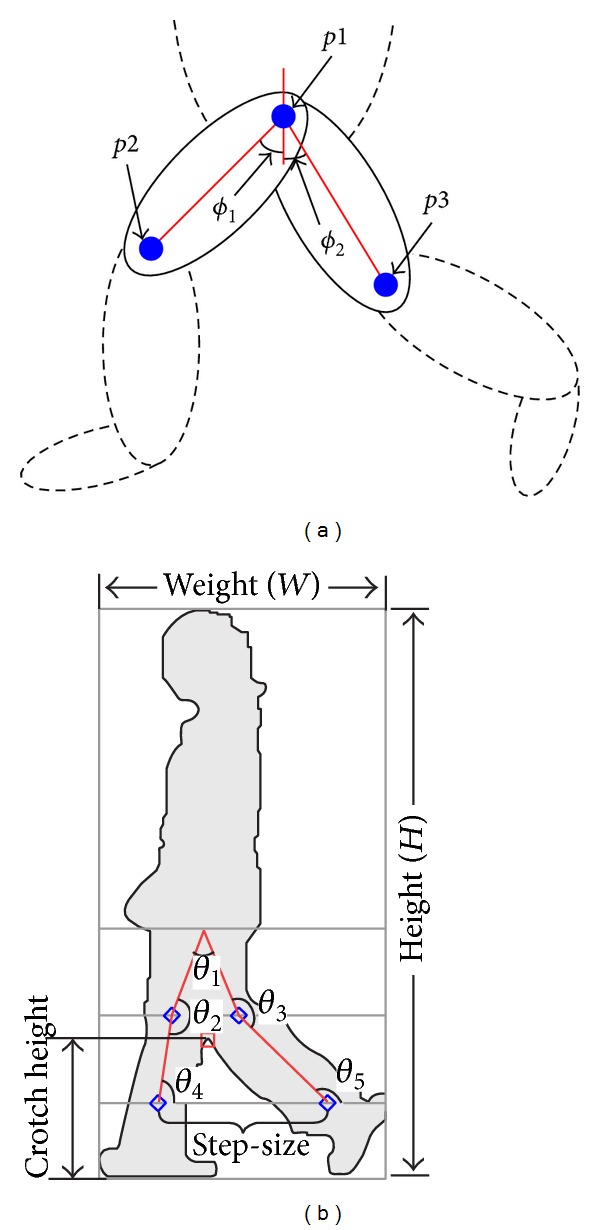
(a) Computation of joint angular trajectory. (b) Nine extracted gait features.

Figure 8(a) illustrates in what manner the joint angular trajectory is determined from two joints. The joint angular trajectory (*θ*) can be determined by using the following equation:

(7) $$\begin{array}{c} \phi_{1}=\text{ta}{\text{n}}^{-1}\left(\frac{p2_{x}-p1_{x}}{p2_{y}-p1_{y}}\right),\\ \phi_{2}=\text{ta}{\text{n}}^{-1}\left(\frac{p3_{x}-p1_{x}}{p3_{y}-p1_{y}}\right),\;\;\theta =\phi_{1}+\phi_{2}, \end{array}$$

where *p*1_*x*_, *p*2_*x*_, and *p*3_*x*_ are the *x*-coordinates of joint *p*1, *p*2, and *p*3, respectively, and *p*1_*y*_, *p*2_*y*_, and *p*3_*y*_ are the *y*-coordinates of joint *p*1, *p*2, and *p*3, respectively.

In our system, five joint angular trajectories have been extracted, as there are the five main joints on the limbs. These angular trajectories are hip angular trajectory (*θ*_1_), front knee angular trajectory (*θ*_2_), back knee angular trajectory (*θ*_3_), front ankle angular trajectory (*θ*_4_), and back ankle angular trajectory (*θ*_5_).

The Euclidean distance between the ankles is used to represent the subject's step-size (*S*). Then, the Euclidean distance between the ground and the subject's crotch is being calculated as crotch height (CH). If the crotch height is found lower than the height of knee, we will assumed that it is equal to zero, as the crotch is considered occluded.  Figure 8(b) shows a sample of a human silhouette with the extracted nine gait features.

To construct the feature vector, maximum hip angular trajectory (*θ*_1_^max⁡^) was determined during a walking sequence. When *θ*_1_^max⁡^ was identified, the corresponding *S*, *W*, *H*, *θ*_2_, *θ*_3_, *θ*_4_, *θ*_5_, and CH were also determined. To better describe the human gait, 24 features were used to construct the feature vector as shown:

(8) F={θ1max⁡,S,W,H,θ2,θ3,θ4,θ5,CH,AW,AH,  ACH,Aθ1,Aθ2,Aθ3,Aθ4,Aθ5,AS,  RAH,RACH,RAS,RCH,RH,RS},

where *A* ^*W*^, *A* ^*H*^, *A* ^CH^, *A* ^*θ*_1_^, *A* ^*θ*_2_^, *A* ^*θ*_3_^, *A* ^*θ*_4_^, *A* ^*θ*_5_^, and *A* ^*S*^ are the average of the local maxima detected for width, height, crotch height, hip angular trajectory, front knee angular trajectory, back knee angular trajectory, front ankle angular trajectory, back ankle angular trajectory, and step-size, respectively; *R* ^AH^, *R* ^ACH^, *R* ^AS^, *R* ^CH^, *R* ^*H*^, and *R* ^*S*^ are the ratio of *A* ^*H*^, *A* ^CH^, *A* ^*S*^, CH, *H*, and *S* to *W*, respectively.

### 3.6. Features of Smoothing and Normalization

As the presence of outliers in the extracted features would hinder the classification process, Gaussian filter with sigma values (*σ*) equal to 2.5 is applied to remove them. In order to normalize the extracted features from various dimensions to be independent and standardized, linear scaling technique [25] has been applied to normalize each feature component to the range between 0 and 1.

### 3.7. Features Selection

The performance of a recognition system is determined by the effectiveness of the selected features, which can maximize interclass variance. In other contexts, the redundant and inappropriate features which degrade the classification rate would be found and removed.

**Figure 9:**
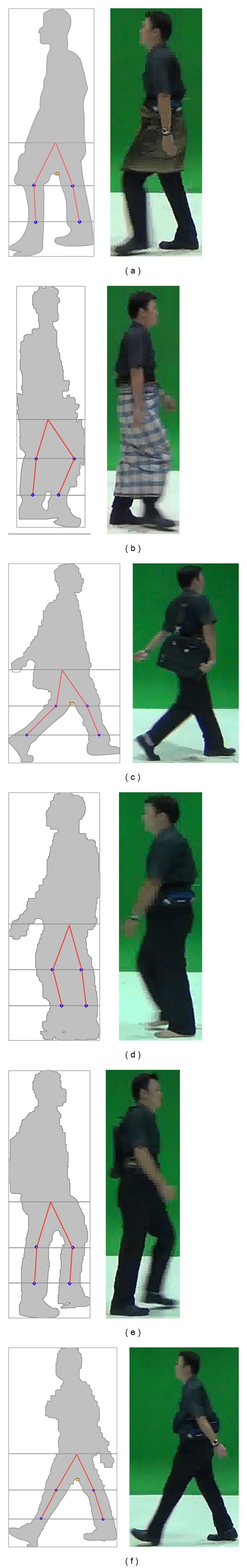
Occluded human silhouettes with joints detection. (a) Wearing “kain samping”. (b) Wearing “sarong”. (c) Barrel bag slung over shoulder. (d) Walking in bare feet. (e) Carrying rucksack. (f) Carrying barrel bag by hand.

In the proposed system, Ranker algorithm proposed by Hall et al. [26] is used to rank features by their individual evaluations, which helps to identify those extracted features that contribute positively in the recognition process. Based on the scores obtained, all twenty-four features have exhibited positive contribution. Thus, all of them are used in our system.  Figure 9 shows examples of successful joint detection from self-occluded silhouettes and silhouettes with external occlusion.

### 3.8. Classification Techniques

To study the performance of our gait recognition system, four classification techniques were applied to find the best correct classification rate and to verify the consistency of the results. In our work, multiclass Support Vector Machine (SVM), Back-propagation artificial neural network (BPANN), Fuzzy k-nearest neighbor (Fuzzy k-NN) with Euclidean distance metrics, and Linear Discriminant Analysis (LDA) classifiers are employed.

For SVM, experiments were carried out to examine the effects on kernel functions—Linear (Ln), Polynomial (Poly), and Radial Basis Function (RBF). The kernel's parameters such as *d* (degree), *g* (gamma), and *r* (coefficient) and regularization parameter *C* were trained to find the best correct classification rate. For Fuzzy k-NN, numerous numbers of neighbors *k* have been tested. For BPANN, various numbers of hidden layers have been trained to find the best classification rate.

As cross-validation procedure is essential to evaluate the accuracy of the classification performance. We employed tenfold cross-validation for this project, where the walking sequences from the gait databases were randomly divided into ten disjoint subsets, nine subsets used for analysis training and one subset used for validation. The cross-validation process was iterated for 10 turns with features vectors of each disjointed subset channeled into classifiers as the validation test. Then, the single mean correct classification rate can be obtained by averaging the cross-validation results.

The experiments were carried out on four classification techniques with various optimization parameters that were obtained during the training. Three quality measures were used in the experiment: correct classification rate (CCR), true positive rate (TPR), and false positive rate (FPR).

## 4. Experimental Results and Discussion

In order to evaluate the performance of the proposed gait recognition system on the new database, numerous experiments have been conducted. This section presents and discusses the results of these experiments which were aimed to assess the recognition rate of the proposed system with respect to view normalization, large population, and covariate factors.

Three databases were employed for performance evaluation; MMUGait Large DB, MMUGait Covariate DB and one database from SOTON Small DB for comparison. In the evaluation of each database, the analysis is performed on walking sequences captured from side-view (Side), normalized oblique-view (NorOb) and a combination of both views (Com). All walking sequences from three databases were used during the training and testing stages.

For group covariate factor analysis, the walking sequences were categorized into five groups: Group 1 (G1)—different speeds; Group 2 (G2)—variety of shoes; Group 3 (G3)—various objects carrying; Group 4 (G4)—various types of apparel; Group 5 (G5)—personal clothing without carrying any object.

### 4.1. Experimental Results of MMUGait Large DB

**Figure 10:**
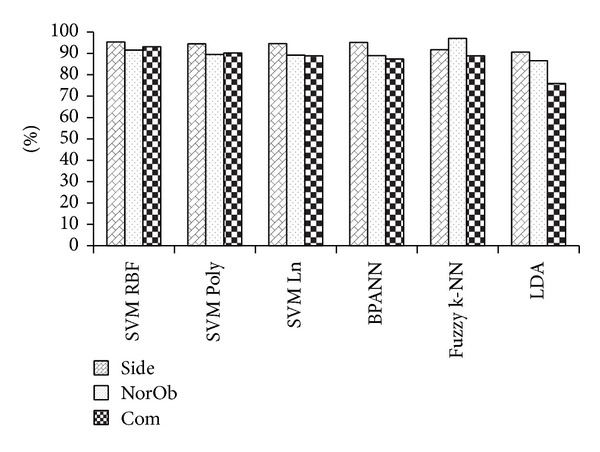
CCRs of MMUGait Large DB.

The performance is evaluated on 80 subjects from MMUGait Large DB. The side-view consists of 2961 walking sequences, the normalized oblique-view consists of 2843 walking sequences and the combination of both views will give a total of 5804 walking sequences. The overall CCR results are summarized in Figure 10. The best overall performance came from SVM RBF with CCRs of 95.4%, 91.6% and 93.3% for side-view, normalized oblique-view and combination of both views, respectively.

### 4.2. Experimental Results of MMUGait Covariate DB

**Figure 11:**
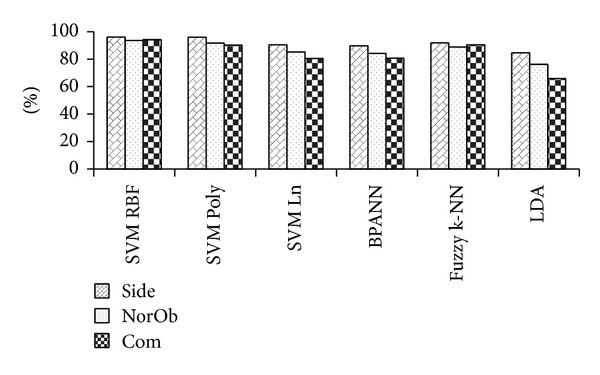
CCRs of MMUGait Covariate DB.

The performance is evaluated on 19 subjects from MMUGait Covariate DB. The side-view consists of 3780 walking sequences, normalized oblique-view consists of 3713 walking sequences, and the combination of both views will give a total of 7493 walking sequences. The overall CCR results are summarized in Figure 11. In overall, the best performance came from SVM RBF with CCRs of 96.0%, 93.6%, and 94.2% for side-view, normalized oblique-view and, combination of both views, respectively.

**Figure 12:**
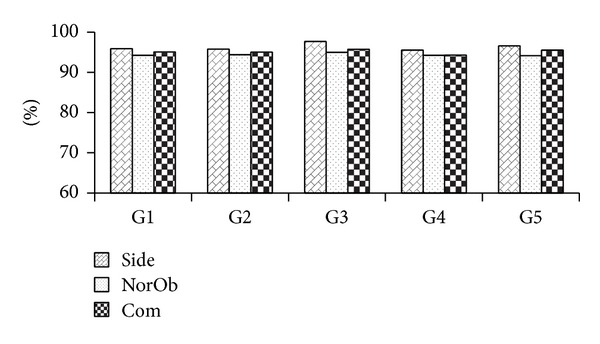
CCRs of group covariate factors for MMUGait Covariate DB.

**Figure 13:**
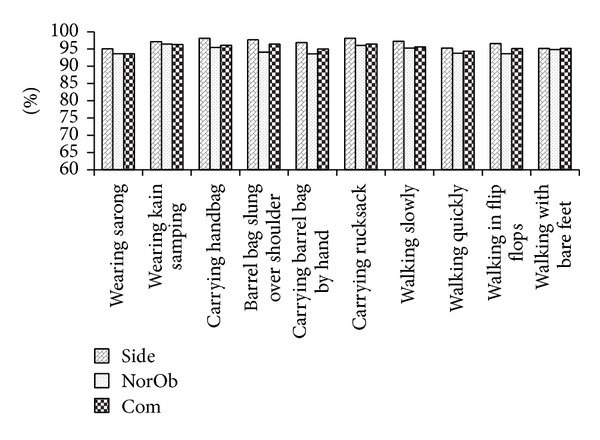
CCRs of individual covariate factor for MMUGait Covariate DB.

Spurred by the encouraging results, subsequent experiments using SVM RBF were conducted to evaluate the system on various covariate factors. The overall CCRs for group and individual covariate factor are summarized in Figures 12 and 13, respectively.

### 4.3. Experimental Results of SOTON Small DB

**Figure 14:**
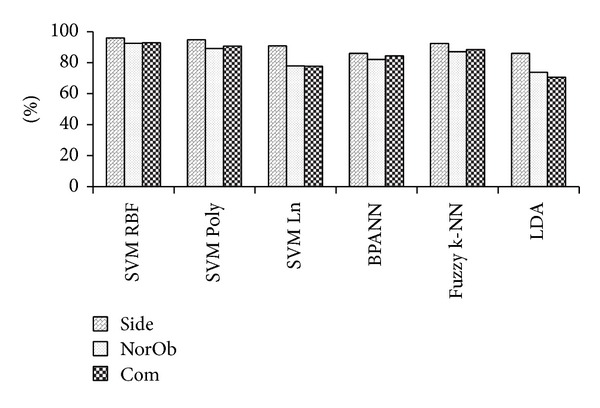
CCRs of SOTON Small DB.

The performance is evaluated on the complete 11 subjects from SOTON Small DB [2]. The side-view consists of 3178 walking sequences, normalized oblique-view consists of 3036 walking sequences, and the combination of both views will give a total of 6214 walking sequences. The overall CCR results are summarized in Figure 14. The best overall performance came from SVM RBF with CCRs of 96.0%, 92.5%, and 92.9% for side-view, normalized oblique-view, and combination of both views, respectively.

**Figure 15:**
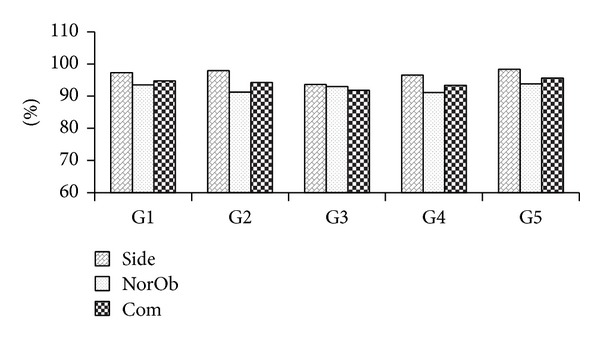
CCRs of group covariate factors for SOTON Small DB.

**Figure 16:**
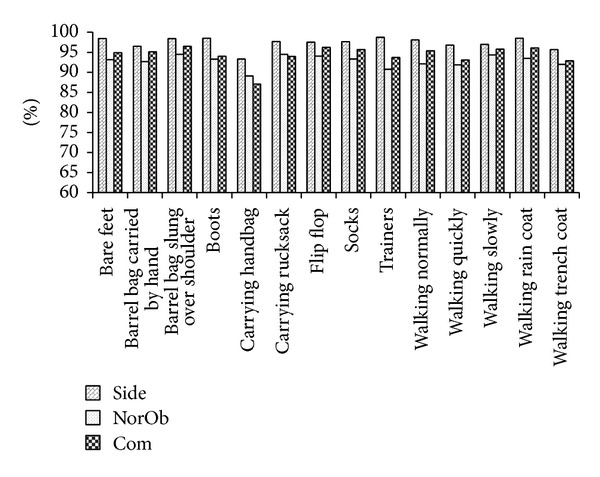
CCRs of individual covariate factor for SOTON Small DB.

Motivated by the encouraging results, subsequent experiments using SVM RBF were conducted to evaluate the performance on multiple covariate factors. The overall CCR results for group and individual covariate factor are summarized in Figures 15 and 16, respectively.

### 4.4. Results Discussion

In general, our gait recognition system managed to obtain high CCRs in all the experiments. All the highest CCRs from each experiment are above 90%. The TPRs obtained are identical to the CCRs. The system achieved low FPRs, which are in the range of 0.1% to 2.6%.

From Figures 12 and 15, it can be observed that our system is robust to covariant factors as it has resulted in high CCRs. For that reason, we found that Group G1 generated high CCR as the duration of the walking cycle was not included as a feature. Similarly, the CCRs generated from group G2 and G3 were high as well. This showed that shoe and bags did not affect the extracted feature. In contrast, group G4 generated lower CCRs; this was because of the crotch height that was unidentified due to the occlusion by apparel. Group G5 presented the best results as there was no covariate factor involved.

From Figures 13 and 16, it can be observed that our system is able to provide high CCRs even when the subjects were wearing long fabrics or carrying objects. However, the clothing factor (“sarong”) in MMUGait Covariate DB and the objects carrying factor (carrying handbag) in SOTON Small DB have resulted the lowest CCRs among other covariates factors. This is mainly because it was not possible to measure the crotch height of the subject due to the occlusion by clothing and bag.

In general, SVM outperforms all the other three classification techniques. In particularly, the nonlinear SVM with RBF or Poly kernel outperforms SVM with Ln kernel. As our generated gait feature vectors are not linear, the kernel trick in the non-linear SVM permits the algorithm to adapt the maximum-margin hyperplane in a transformed feature space [27]. As expected, the RBF kernel produces better results as it normally reigned over Poly kernel [28]. Overall, nonlinear SVM outperforms BPANN, as the BPANN suffers from the existence of multiple local minimal solutions. However, the solution of SVM is unique and global [29].

LDA performed poorer than the other three classification techniques. This may be due to the reason that the hyperplane computed by LDA is only optimal when the covariance matrices for all of the classes are identical [30], whereas the performance of Fuzzy k-NN was stable and it was the second best among the four techniques.

To the best of our knowledge, no other researcher has published results on oblique-view SOTON Small DB. We can only compare finding on side-view SOTON Small DB.  Table 1 shows the comparison of our best experiment results (SVM with RBF) with other gait recognition systems in detail.

From Table 1, the highest CCR (95.97%) obtained outperforms the results obtained by Bouchrika and Nixon [7] and Pratheepan et al. [11]. The poorer result by Bouchrika and Nixon is due to the requirement to manually label model template to describe joints' motion. Conversely, our results are better than Pratheepan et al., as we do not incorporate the selection or estimation of gait cycle. Furthermore, we are the only group that have tested the complete database with 11 subjects, 15 covariate factors, and 3178 walking sequences compared with Bouchrika and Nixon (10 subjects, 11 covariate factors, and 440 walking sequences), Pratheepan et al. (10 subjects, 4 covariate factors, and 180 walking sequences), and Bashir et al. (11 subjects, unknown number of covariate factors, and 373 walking sequences).

Even though Bashir et al. [9] produced better results, it is inappropriate to compare our results with them as they have only tested about 10% of the full walking sequences and the total numbers of the covariate factors being employed is unclear.

## 5. Conclusion

We presented an automated multiview gait recognition system by employing hybrid approach (model-free and model based). It was tested on a new gait database, MMUGait which consists of new variations in clothing. In the proposed approach, the joint angular trajectories can be computed from the detected body joints even on self-occluded or external occluded silhouettes. Thus, it has shown to be more effective than other approaches in related research.

In addition, the high CCRs and TPRs and low FPRs also show that it is robust and can achieve good performance either in gait databases with various covariate factors or large population of subjects and multiple view angles. For upcoming research, more gait databases will be tested by the proposed approach to evaluate its performance.

The authors are planning to allow public access to the MMUGait database in the near future. We believe that the walking sequences with special apparels will be invaluable for performance evaluation of other gait recognition systems.

## Figures and Tables

**Table 1:** Comparison with other systems employing SOTON Small DB.

	Bashir et al. [9]	Bouchrika and Nixon [7]	Pratheepan et al. [11]	Our approach
CCR (%)	97.1	73.4	86.0	96.0
Number of subjects tested	Eleven	Ten	Ten	Eleven
Number of covariate factors tested	Unspecified	Twelve	Four	Fifteen
Number of walking sequences tested	373	440	180	3178

## References

[1] Little J, Boyd J (1998). Recognizing people by their gait: the shape of motion. *Videre: Journal of Computer Vision Research*.

[2] Shutler J, Grant M, Nixon MS, Carter JN On a large sequence-based human gait database.

[3] Sarkar S, Phillips PJ, Liu Z, Vega IR, Grother P, Bowyer KW (2005). The humanID gait challenge problem: data sets, performance, and analysis. *IEEE Transactions on Pattern Analysis and Machine Intelligence*.

[4] Yu S, Tan D, Tan T A framework for evaluating the effect of view angle, clothing and carrying condition on gait recognition.

[5] Makihara Y, Hidetoshi M, Akira T (2012). The OU-ISIR gait database comprising the treadmill dataset. *IPSJ Transactions on Computer Vision and Applications*.

[6] Lee SJ, Hidler J (2008). Biometrics of overground vs. treadmill walking in healthy individuals. *Journal of Applied Physiology*.

[7] Bouchrika I, Nixon MS Exploratory factor analysis of gait recognition.

[8] Cunado D, Nixon MS, Carter JN (2003). Automatic extraction and description of human gait models for recognition purposes. *Computer Vision and Image Understanding*.

[9] Bashir K, Xiang T, Gong S, Mary Q (2009). Gait representation using flow fields. *British Machine Vision Conference*.

[10] Goffredo M, Bouchrika I, Carter JN, Nixon MS (2010). Self-calibrating view-invariant gait biometrics. *IEEE Transactions on Systems, Man, and Cybernetics B: Cybernetics*.

[11] Pratheepan Y, Condell JV, Prasad G, Fritz, Schiele B M, Piater JH (2009). Individual identification using Gait sequences under different covariate factors. *Computer Vision Systems*.

[12] Yoo JH, Nixon MS (2011). Automated markerless analysis of human gait motion for recognition and classification. *ETRI Journal*.

[13] Dempster WT, Gaughran G (1967). Properties of body segments based on size and weight. *The American Journal of Anatomy*.

[14] Bouchrika I, Goffredo M, Carter J, Nixon MS, Tistarelli M, Nixon MS (2009). Covariate analysis for view-point independent Gait recognition. *Advances in Biometrics*.

[15] Jean F, Albu AB, Bergevin R (2009). Towards view-invariant gait modeling: computing view-normalized body part trajectories. *Pattern Recognition*.

[16] Kale A, Chowdhury AKR, Chellappa R Towards a view invariant Gait recognition algorithm.

[17] Lee CS, Elgammal A, Kanade T, Jain A, Ratha NK (2005). Towards scalable view-invariant Gait recognition, multilinear analysis for Gait. *Audio-and Video-Based Biometric Person Authentication*.

[18] Bodor R, Drenner A, Fehr D, Masoud O, Papanikolopoulos N (2009). View-independent human motion classification using image-based reconstruction. *Image and Vision Computing*.

[19] Shakhnarovich G, Lee L, Darrell T Integrated face and gait recognition from multiple views.

[20] Kusakunniran W, Wu Q, Li H, Zhang J Multiple views Gait recognition using view transformation model based on optimized Gait energy image.

[21] Makihara Y, Sagawa R, Mukaigawa Y, Echigo T, Yagi Y, Leonardis A, Bischof H, Pinz A (2006). Gait recognition using a view transformation model in the frequency domain. *Computer Vision*.

[22] Murray MP (1967). Gait as a total pattern of movement. *The American Journal of Physical Medicine*.

[23] Ho CC, Ng H, Tan WH (2013). MMU GASPFA: a COTS multimodal biometric database. *Pattern Recognition Letters*.

[24] Otsu N (1979). A threshold selection method from gray-level histograms. *Automatica*.

[25] Aksoy S, Haralick RM (2001). Feature normalization and likelihood-based similarity measures for image retrieval. *Pattern Recognition Letters*.

[26] Hall M, Frank E, Holmes G, Pfahringer B, Reutemann P, Witten I (2009). The WEKA data mining software: an update. *ACM SIGKDD Explorations Newsletter*.

[27] Suykens J, Gestel V, Brabanter JD, Moor BD, Vandewalle J (2002). *Least Squares Support Vector Machines*.

[28] Byun H, Lee SW (2003). A survey on pattern recognition applications of support vector machines. *International Journal of Pattern Recognition and Artificial Intelligence*.

[29] Schölkopf B, Burges C (1999). *Advances in Kernel Methods: Support Vector Learning*.

[30] Gokcen I, Peng J, Yakhno T (2002). Comparing linear discriminant analysis and support vector machines. *Advances in Information Systems*.

